# Vanillic acid alleviates methamphetamine-induced mitochondrial toxicity in cardiac mitochondria via antioxidant activity and inhibition of MPT Pore opening: an in-vitro study

**DOI:** 10.1186/s40360-023-00676-9

**Published:** 2023-05-19

**Authors:** Mohammad Shabani, Zhaleh Jamali, Deniz Bayrami, Ahmad Salimi

**Affiliations:** 1 Students Research Committee, Faculty of Pharmacy, Ardabil University of Medical Sciences, Ardabil, Iran; 2 Traditional Medicine and Hydrotherapy Research Center, Ardabil University of Medical Sciences, Ardabil, Iran; 3 Department of Pharmacology and Toxicology, School of Pharmacy, Ardabil University of Medical Sciences, Ardabil, Iran; 4 Department of Addiction Studies, School of Medicine, Shahroud University of Medical Sciences, Shahroud, Iran; 5 Student Research Committee, School of Medicine, Shahroud University of Medical Sciences, Shahroud, Iran; 6 Toxicology and Pharmacology School of Pharmacy, Ardabil University of Medical Sciences, P.O. Box: 56189-53141, Ardabil, Iran

**Keywords:** Polyphenolic Compounds, Cardioprotective, Illicit drugs, Amphetamine, Cardiomyopathy

## Abstract

**Background:**

Methamphetamine is widely abused in all parts of the world. It has been reported that short-term and long-term methamphetamine exposure could damage the dopaminergic system and induce cardiomyopathy and cardiotoxicity via mitochondrial dysfunction and oxidative stress. Vanillic acid (VA), a phenolic acid compound derived from plants, is known for its antioxidant and mitochondrial protection properties.

**Methods:**

In the current study we used VA for attenuating of Methamphetamine-induced mitochondrial toxicity in cardiac mitochondria. Isolated mitochondria obtained from rat heart were grouped as: control, methamphetamine (250 µM), VA (10, 50 and 100 µM) was cotreated with methamphetamine (250 µM) and VA (100 µM) alone. After 60 min, mitochondrial fraction including: succinate dehydrogenases (SDH) activity, mitochondrial membrane potential (MMP), mitochondrial swelling, mitochondrial glutathione (GSH), reactive oxygen species (ROS) and lipid peroxidation (LPO) were evaluated.

**Results:**

Methamphetamine exposure significantly disrupted mitochondrial function and induced ROS formation, lipid peroxidation, GSH depletion, MMP collapse and mitochondrial swelling, while VA significantly increased SDH activity as indicator of mitochondrial toxicity and dysfunction. VA also significantly decreased ROS formation, lipid peroxidation, mitochondrial swelling, MMP collapse and depletion of GSH in cardiac mitochondria in the presence of methamphetamine.

**Conclusion:**

These findings suggested that VA is able to reduce methamphetamine-induced mitochondrial dysfunction and oxidative stress. Our results demonstrate that VA could potentially serve as a promising and accessible cardioprotective agent against methamphetamine-induced cardiotoxicity, via antioxidant and mitochondrial protection properties.

## Background

Methamphetamine as a major drug of abuse is used in many countries (in 2021, over 2.5 million people in the United States) and its use is associated with adverse health effects on multiple organ systems such as heart. The use and overdose of methamphetamine leads to arrests and deaths [1, 2]. Methamphetamine as a psychostimulant drug has two enantiomers D-methamphetamine and L-methamphetamine [3]. Mechanistically, due to structural similarity methamphetamine to catecholamine neurotransmitters (dopamine, epinephrine, norepinephrine and serotonin), this cationic lipophilic molecule can substitute for the serotonin transporter (SERT), noradrenaline transporter (NET), vesicular monoamine transporter-2 (VMAT-2) and dopamine transporter (DAT) and reverse their endogenous function, resulting in redistribution monoamines from vesicles into the cytosol [3]. This substitution results in the release of catecholamine neurotransmitters into the synapse and stimulation of postsynaptic monoamine receptors [3]. Methamphetamine also partially blocks the reuptake of catecholamines in the central nervous system (CNS) and attenuates the metabolism of them by inhibiting monoamine oxidase, leading to the buildup of overplus monoamines in the synapse [3]. The catecholamine neurotransmitters released due to the presence of methamphetamine act on the major serotonergic, noradrenergic and dopaminergic pathways of the brain and other part of body [4]. Due to the wide distribution of monoamines receptors throughout the body methamphetamine can affect several organ systems, but the most clinically observed effects are cardiovascular and neurologic [5]. Previous studies have reported cardiovascular effects such as direct vasospasm or vasoconstriction, fluctuation in reactive oxygen species (ROS) formation, induction of inflammation, reduced NO-mediated vasodilation, blood pressure (BP), elevated heart rate (HR) and myocardial contractility after the use of methamphetamine [6]. Autopsy and clinical findings obtained from long-term methamphetamine users demonstrated indicators of cardiomyopathy, such as enlargement of the heart, hypertrophy, necrosis and fibrosis [7]. Methamphetamine-related cardiotoxic effects have been reported in animal studies and were associated with intracellular and extracellular edema disarray of cardiomyocytes, myocyte degeneration, dilated T tubules, contraction band degeneration, myofilament loss, abnormally shaped nuclei and mitochondria [8]. Studies have been shown that both short-term and longer-term methamphetamine administration can induce hypertrophy and cellular damage in cultured, isolated cardiomyocytes and support a role for catecholamine-independent direct cardiotoxicity. Exposure to methamphetamine promotes mitochondrial dysfunction, ROS generation, myocardial ischemia metabolic dysregulation and cardiomyopathy [6]. In a mouse model, methamphetamine exposure led to impaired oxidative phosphorylation (OXPHOS) and a decrease in the expression of mitochondrial protein FIS1 [9]. A recent study in cardiac isolated mitochondria has revealed that methamphetamine causes a deleterious changes in mitochondrial functions, ROS formation, MMP collapse, mitochondrial swelling, lipid peroxidation and oxidative stress [10]. Mitochondria have a key role in myocardial tissue homeostasis; thus, mitochondrial dysfunction eventually leads to endothelial cell and cardiomyocyte death and consequent cardiovascular disorders [11, 12]. Therefore, based on methamphetamine’s mechanism of action on mitochondria, methamphetamine can damage to mitochondria and induce cardiotoxicity via oxidative stress and mitochondrial dysfunction. Probably a promising strategy to reduce methamphetamine-induced cardiovascular damage is the use of mitochondrial protective agents and antioxidants. These agents can protect mitochondria in myocardial tissue and inhibit mitochondrial dysfunction leading to cardiomyocyte death and cardiotoxicity [13].

Natural compounds have become the main target for investigators to study the protective/ therapeutic effect to fight against drug and chemical-induced cardiotoxicity via mitochondria dysfunction and oxidative stress [14, 15]. A growing body of research has shown that natural compounds exert anti-apoptotic effects on drug and chemical-induced cardiotoxicity via mitochondrial protection and reduction of oxidative stress [16]. Therefore, a remarkable attention has been paid to polyphenolic compounds [17]. Vanillic acid (VA) or 4-hydroxy-3-methoxybenzoic acid is one of the antioxidants derived from whole grains, herbs, juices, fruits, wines and beers, which exerts diverse bioactivity against diabetes, cancer, obesity, neurodegenerative, hepatic and cardiovascular diseases [18]. ROS formation and oxidative stress have the key role in causing the mentioned diseases where VA can improve these disorders via its antioxidant potential [19, 20]. In addition, various studies have demonstrated that VA possesses diverse pharmacological activities such as anti-inflammatory, antioxidant, antihypertensive, antimicrobial and inhibition of snake venom activity [18]. Based on previous studies VA reduces ROS production and lipid peroxidation, improves mitochondrial function, scavenges free radicals, enhances antioxidative status, thereby decreasing cardiac dysfunction [21, 22]. Therefore, in our current study, we have formulated a hypothesis that VA could potentially protect the harmful effects of methamphetamine on cardiac mitochondria. This hypothesis is based on our understanding of methamphetamine’s mechanism of action on mitochondria and the critical role that these organelles play in the functioning of cardiomyocytes. For this purpose, the ameliorative effect of VA in the presence of methamphetamine was investigated in cardiac mitochondria, and after 60 min, mitochondrial fraction including: SDH activity, MMP collapse, mitochondrial swelling, mitochondrial GSH, ROS formation and LPO were evaluated.

## Martials and methods

### Chemicals

Magnesium chloride, Potassium chloride, Butylated Hydroxytoluene (BHT), Bovine serum albumin (BSA), Rhodamine 123, D mannitol, Ethylenediaminetetraacetic acid (EDTA), 2´,7´-Dichlorofluorescein (H2DCF), Monopotassium phosphate, Ethylene glycol-bis(b-aminoethyl ether (EGTA), Coomassie Brilliant Blue, Rotenone, Sucrose, 4,5-dimethylthiazol-2-yl)-2,5-diphenyltetrazolium bromide (MTT),2-Amino-2-hydroxymethyl- propane-1,3-diol (TRIS), 3-morpholinopropane-1-sulfonic acid (MOPS), Sodium succinate, Dimethyl sulfoxide (DMSO), N-(2-hydroxyethyl) piperazine-N 0-(2-ethanesulfonic acid) (HEPES) and Vanillic Acid with purity of about 99% and 121-34-6 was purchased from Sigma (St. Louis, MO USA). Methamphetamine with CAS number 51–57 − 0 and purity of about 99%, was gifted from School of Medicine, Shahroud University of Medical Sciences (Shahroud, Iran). It was freshly prepared before use and dissolved in deionized water. Vanillic acid was freshly prepared before the use and dissolved in DMSO (0. 5%).

### Animals

Male Wistar rats (220 g, 8 weeks old, total number of animals = 5) were purchased from the Pasteur Institute of Iran (Tehran, Iran). The animals were housed in the animal house of the Faculty of Pharmacy, Ardabil University of Medical Sciences for 14 days under a 12-h/12-h light/dark cycle at 25 ± 2 °C with 50 ± 20% humidity. They were provided with water ad libitum and food. All of the experimental procedures were conducted according to the guidelines of the Ethics Committee of the Ardabil University of Medical Sciences (Approval ID: IR.ARUMS.AEC.1400.021).

### Isolation of cardiac mitochondria

The animals were anesthetized with mixture of ketamine (70 mg/kg) and xylazine (3 mg/kg) and after a deep anesthesia were sacrificed by cervical dislocation. The hearts were quickly removed and used for obtaining cardiac mitochondria. We isolated cardiac mitochondria from the whole heart using a standard technique, as previously described [10, 23]. The hearts were separately chopped, freed of blood vessels, and mechanically homogenized with a glass homogenizer in a tenfold volume of isolation buffer containing 75 mM sucrose, 225 mM D-mannitol and 0.2 mM EDTA, pH = 7.4. The homogenate was centrifuged at 1000× g for 10 min, and the pellet was discarded to remove cell debris and nuclei. Then the supernatant was sedimented at 10,000× g for 10 min. All methods were performed at 4 °C on ice. We determined the mitochondrial protein content using the Bradford assay [24]. The protein concentration used for each test was 1000 µg/ml.

### Estimation of mitochondrial succinate dehydrogenase activity

The in vitro mitochondrial toxicity and ameliorative effect of VA against methamphetamine was determined by MTT assay [10]. Briefly, 100 µL of the mitochondrial suspension (100 µg/well) in mitochondrial assay buffer (2 mmol/L MgCl_2_, 0.5 mmol/L KH_2_PO_4_, 10 mmol/L NaCl, 140 mmol/L KCL, 0.5 mmol/L EGTA, 20 mmol/L HEPES; supplemented with 10 mmol/L succinate and1mg/mL rotenone, pH = 7.4), were added to 96-well plates, then 4 µL of stock solution of DMSO (13%), 4 µL of stock solution of methamphetamine (6.5 mM), 2 µL of stock solution of methamphetamine (13 mM) + 2 µL of stock solution of VA (520, 2600 and 5200µM) and 4 µL of stock solution of VA (2600 µM) were added to control, methamphetamine (250 µM), methamphetamine (250 µM) + VA (10 µM), methamphetamine (250 µM) + VA (50 µM), methamphetamine (250 µM) + VA (100 µM) and VA (100 µM) groups respectively. Cardiac mitochondria were placed in a humidified 5% CO_2_ incubator for 60 min at 37 °C. After incubation, 25 µL of MTT solution (0.5 mg/ml in mitochondrial assay buffer) were added to each well and re-incubated for 30 min at 37 °C. Then, the 100 µL of DMSO were carefully were added to each well to dissolve the formazan crystals. The plate was placed on a Biotek microplate reader and the absorbance was measured at 570 nm. All assays were performed in three replicates. SDH activity was calculated as the percentage of MTT absorption as follows: % SDH activity = (mean experimental absorbance/mean control absorbance×100).

### Estimation of mitochondrial membrane potential

To analyze the alterations of MMP in cardiac mitochondria after the exposure to methamphetamine and VA, the fluorescence intensity of rhodamine was measured using flow cytometer. Briefly, cardiac mitochondria were added into 24-well plate with a density of 1000 µg/ml in MMP buffer (10 mM HEPES, 220 mM sucrose, 68 mM D-mannitol, 50 µM EGTA, 2 mM MgCl_2_, 5 mM KH_2_PO_4_, 5 mM sodium succinate, 10 mM KCl and 2 µM rotenone, pH = 7.4) Then, cardiac mitochondria were exposed to methamphetamine (250 µM), methamphetamine + VA (10 µM), methamphetamine + VA (50 µM), methamphetamine + VA (100 µM) and VA (100 µM) for 60 min. After incubation, MMP buffer was replaced with MMP buffer + rhodamine 123 (5 µM) and then incubated in dark for 15 min at room temperature. The mean of fluorescence intensity as reflection of MMP collapse was measured by flow cytometer (Cyflow Space-Partec, Germany) on the FL1 channel.

### Estimation of mitochondrial ROS formation

The mitochondrial ROS level in cardiac mitochondria was determined by H_2_DCF fluorescence dye, as previously described [25]. Briefly, cardiac mitochondria were added into 24-well plate with a density of 1000 µg/ml in respiration buffer (0.5 mM MgCl_2_, 0.32 mM sucrose, 20 mM MOPS, 0.1 mM KH_2_PO_4_, 10 mM Tris, 5 mM sodium succinate, and 50 mM EGTA, pH = 7.4). Then, cardiac mitochondria were exposed to methamphetamine (250 µM), methamphetamine + VA (10 µM), methamphetamine + VA (50 µM), methamphetamine + VA (100 µM) and VA (100 µM) for 60 min. After incubation, respiration buffer was replaced with respiration buffer + H_2_DCF (10 µM) and then incubated in dark for 15 min at room temperature. The mean of fluorescence intensity as reflection of ROS level was measured by flow cytometer (Cyflow Space-Partec, Germany) on the FL1 channel.

### Estimation of mitochondrial glutathione

The content of GSH in cardiac mitochondria was measured by the protocol of Ellman [26]. Briefly, cardiac mitochondria were added into 24-well plate with a density of 1000 µg/ml mitochondrial assay buffer (2 mmol/L MgCl_2_, 0.5 mmol/L KH_2_PO_4_, 10 mmol/L NaCl, 140 mmol/L KCL, 0.5 mmol/L EGTA, 20 mmol/L HEPES; supplemented with 10 mmol/L succinate and 1 mg/mL rotenone, pH = 7.4). Then, cardiac mitochondria were exposed to methamphetamine (250 µM), methamphetamine + VA (10 µM), methamphetamine + VA (50 µM), methamphetamine + VA (100 µM) and VA (100 µM) for 60 min. After incubation, the isolated mitochondria mechanically lysed using glass homogenizer in phosphate buffer (0.1 M with pH = 7.4) and were centrifuged for 8,000 × g at 4 °C for 10 min. 100 µL of supernatant was mixed with 3 ml of reaction solution (10mM of DTNB and 500mM of TRIS–HCl with pH = 8.0). After 15 min of incubation at 25 °C, the yellow color developed was read at 412 nm.

### Estimation of mitochondrial lipid peroxidation

The concentration of malondialdehyde (MDA), as indicator of lipid peroxidation was measured in cardiac mitochondria by the method of thiobarbituric acid (TBA) test. Briefly, cardiac mitochondria were added into 24-well plate with a density of 1000 µg/ml in mitochondrial assay buffer (2 mmol/L MgCl_2_, 0.5 mmol/L KH_2_PO_4_, 10 mmol/L NaCl, 140 mmol/L KCL, 0.5 mmol/L EGTA, 20 mmol/L HEPES; supplemented with 10 mmol/L succinate and 1 mg/mL rotenone, pH = 7.4). Then, cardiac mitochondria were exposed to methamphetamine (250 µM), methamphetamine + VA (10 µM), methamphetamine + VA (50 µM), methamphetamine + VA (100 µM) and VA (100 µM) for 60 min. After incubation, cardiac mitochondria were homogenized mechanically in a tube containing 1 ml 0.1% (w/v) trichloroacetic acid (TCA) by glass homogenizer and centrifugated at 10,000 x g for 10 min. The supernatant was transferred to a tube containing 4 ml of 20% TCA containing 0.5% TBA and for 15 min was kept in a boiling water bath. The absorbance was read at 535 nm.

### Estimation of mitochondrial swelling

The opening of the mitochondrial permeability transition (MPT) pore induces mitochondrial swelling, which can be measured as the decrease in absorbance at 540 nm of the suspension [10]. Briefly, cardiac mitochondria were added into 96-well plate with a density of 100 µg/well in mitochondrial assay buffer (2 mmol/L MgCl_2_, 10 mmol/L NaCl, 0.5 mmol/L KH_2_PO_4_, 140 mmol/L KCL, 20 mmol/L HEPES, 0.5 mmol/L EGTA; supplemented with 10 mmol/L succinate and 1 mg/mL rotenone, pH = 7.4). Then, cardiac mitochondria were exposed to methamphetamine (250 µM), methamphetamine + VA (10 µM), methamphetamine + VA (50 µM), methamphetamine + VA (100 µM) and VA (100 µM) for 60 min. After incubation, the absorbance value of the mitochondrial suspension each fifteen minutes was immediately and continuously recorded at 540 nm. The results were presented as the absorbance decrease at different times compared to those control.

### Statistical analysis

For statistical analysis, the results were expressed as means ± standard deviation (SD) from at least five independent experiments. The statistical differences between the groups were evaluated using the one/two-way analysis of variance (ANOVA) followed by the post hoc Tukey’s and Bonferroni’s test respectively. The difference was considered significant at p < 0.05. The results were analyzed using GraphPad Prism 9 (GraphPad Software, San Diego, CA, United States). FlowJo software was used to analyze the flow cytometry data.

## Results

### VA significantly increases mitochondrial SDH activity

**Fig. 1:**
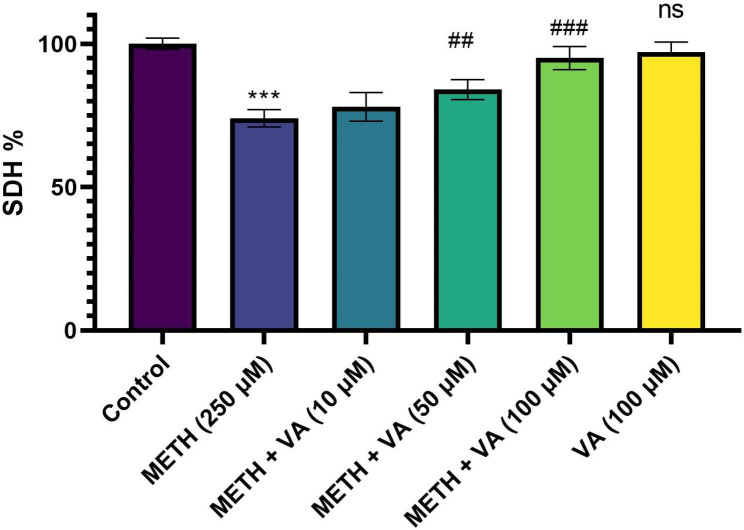
Enhancement of mitochondrial succinate dehydrogenase in cardiac mitochondria by VA in presence of methamphetamine. Cardiac mitochondria were treated/cotreated with methamphetamine, methamphetamine plus various concentrations (10, 50 and 100 µM) of VA and VA (100 µM) alone for 60 min. Mitochondrial succinate dehydrogenase was evaluated by MTT assay. Results were presented as a percentage of control measured in the absence of the compounds. Each point represents the mean ± SD of five independent experiments. *** p < 0.001 versus untreated control; ## p < 0.01 versus methamphetamine group; ### p < 0.001 versus methamphetamine group (one-way ANOVA followed by Tukey’s multiple comparison tests). SDH, succinate dehydrogenase; METH, methamphetamine; VA, Vanillic acid

The activity of mitochondrial SDH was measured as indictor of functionality and integrity of cardiac mitochondria. To determine the effect of methamphetamine (250 µM) and different concentrations of VA (10, 50 and 100 µM) on the mitochondrial SDH activity, 100 µg/well were treated/cotreated with for 60 min. Methamphetamine was toxic against cardiac mitochondria at 250 µM (concentration that inhibits % 26 ± 3.6 of mitochondrial SDH activity during 60 min). As shown in Fig. 1 after 60 min exposure of cardiac mitochondria with various concentrations of VA (50 and 100 µM) and toxic concentration of methamphetamine, mitochondrial SDH activity (percentage) were calculated as 84 ± 4.3, and 95 ± 4.9%, respectively. There were no significant differences in SDH activity of the VA group alone (100 µM) compared to the control group. Based on these data, VA (50 and 100 µM) was significantly able to reduce methamphetamine-induced mitochondrial toxicity. The mean absorbance of the non-exposed cardiac mitochondria was the reference value for calculating 100% SDH activity.

### VA significantly decreases MMP Collapse

**Fig. 2:**
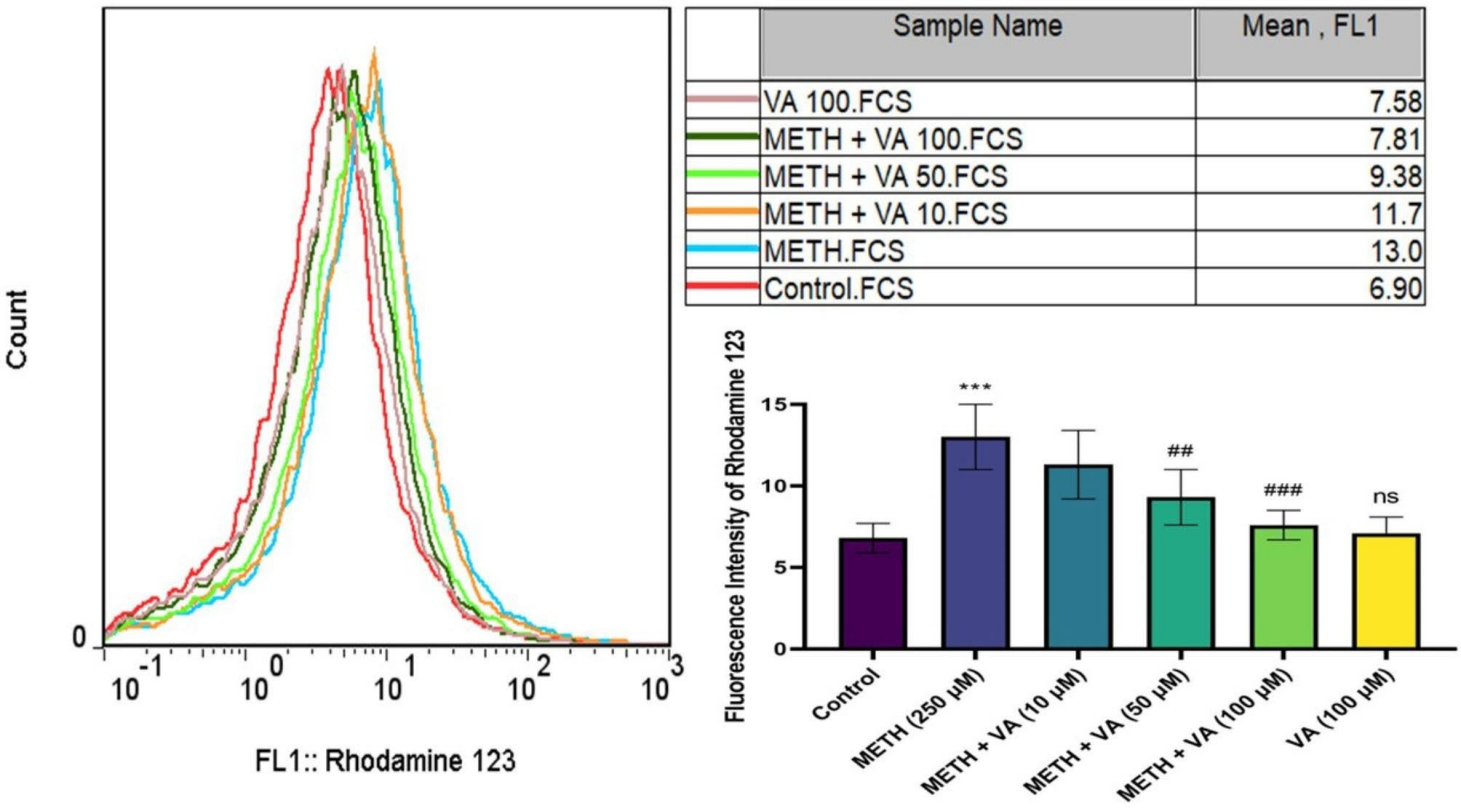
Decline of mitochondrial membrane potential (ΔΨm) collapse in cardiac mitochondria by VA in presence of methamphetamine. Cardiac mitochondria were treated/cotreated with methamphetamine, methamphetamine plus various concentrations (10, 50 and 100 µM) of VA and VA (100 µM) alone for 60 min. ΔΨm was measured using flow cytometric analysis. Treatment with 250 µM methamphetamine increased the ΔΨm. While various concentrations (50 and 100 µM) of VA alleviates methamphetamine-induced ΔΨm. Data are presented as the mean ± SD. *** p < 0.001 versus untreated control; ## p < 0.01 versus methamphetamine group; ### p < 0.001 versus methamphetamine group (one-way ANOVA followed by Tukey’s multiple comparison tests). ΔΨm, mitochondrial membrane potential; METH, methamphetamine; VA, Vanillic acid; SD standard deviation

Mitochondrial function as a key indicator of cell health, can be evaluated by monitoring changes in MMP. To determine the involvement of the MMP collapse (ΔΨm) in mitochondrial protective effects of VA against methamphetamine, the present study detected MMP collapse using flow cytometry, using the cationic membrane potential indicator rhodamine 123. Treatment with 250 µM methamphetamine for 60 min caused the loss of ΔΨm in cardiac mitochondria, compared with the untreated control (Fig. 2). Compared with the methamphetamine group, cotreatment to VA (50 and 100 µM) and methamphetamine for 60 min resulted in a rapid decrease in fluorescence intensity of rhodamine 123 in a dose-dependent manner, which indicated the MMP collapse was restored close to the control group.

### VA significantly decreases mitochondrial ROS formation

**Fig. 3:**
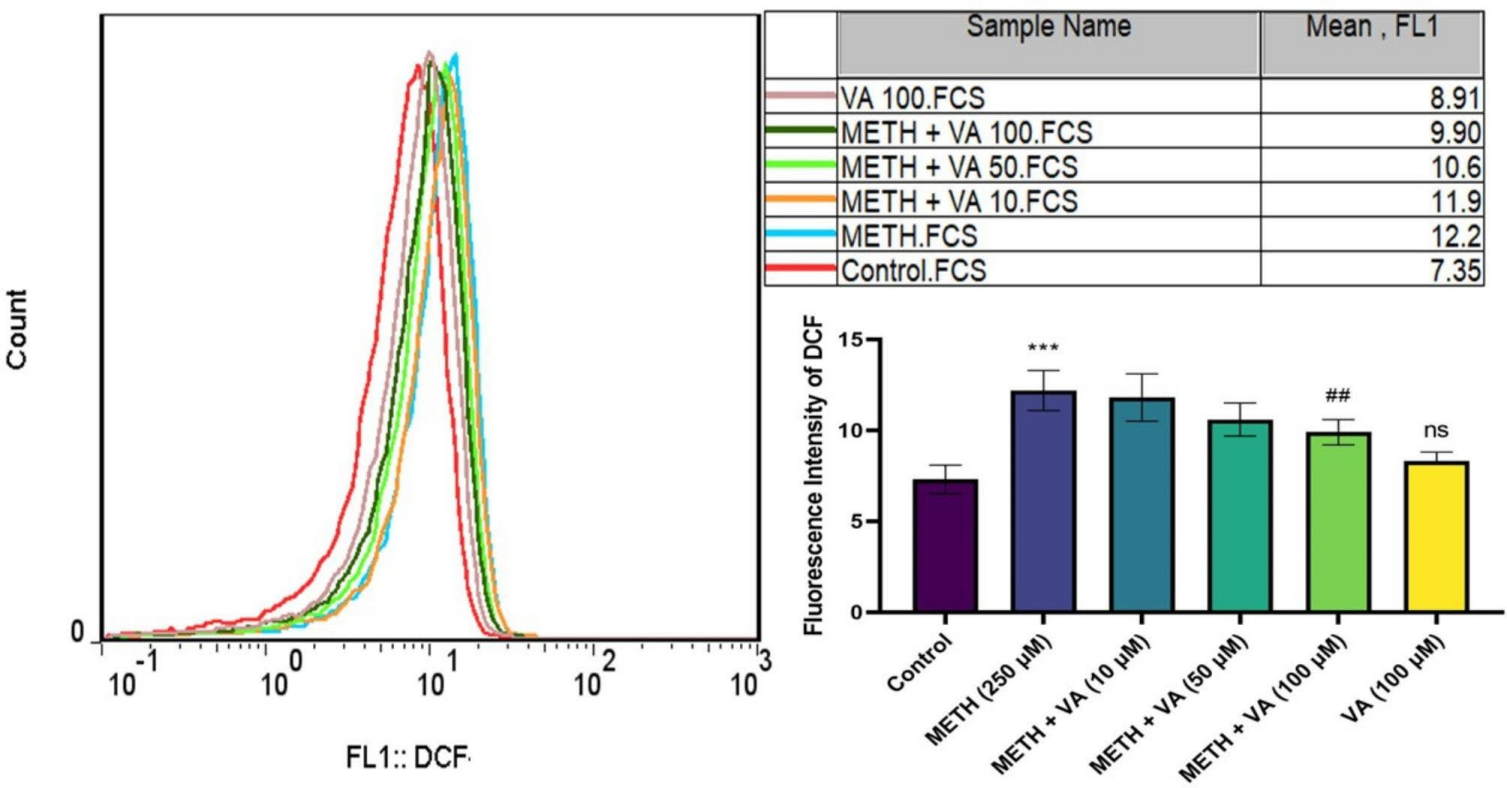
Ameliorative effect of VA on methamphetamine -induced mitochondrial release of ROS. Cardiac mitochondria were treated/cotreated with methamphetamine, methamphetamine plus various concentrations (10, 50 and 100 µM) of VA and VA (100 µM) alone for 60 min. mitochondrial ROS was measured using flow cytometric analysis. Methamphetamine increased mitochondrial ROS in cardiac mitochondria. Treatment with VA (100 µM) significantly ameliorated methamphetamine-induced mitochondrial ROS. Data are presented as the mean ± SD. *** p < 0.001 versus untreated control; ## p < 0.01 versus methamphetamine group (one-way ANOVA followed by Tukey’s multiple comparison tests). ROS, reactive oxygen species; METH, methamphetamine; VA, Vanillic acid; SD standard deviation; DCF, Dichlorofluorescein

ROS formation plays a vital role in cell survival and death. To further examine the VA protective effects on methamphetamine-induced mitochondrial toxicity, the present study investigated its effect on ROS formation in cardiac mitochondria. H_2_DCF, a specific dye for ROS measurement, was used to detect ROS formation. Quantitative measurements of the mean fluorescence intensity of DCF from the methamphetamine group demonstrated that this drug increased mitochondrial ROS generation in cardiac mitochondria (Fig. 3). Whereas, the mitochondrial ROS was markedly decreased following incubation with VA (100 µM) and methamphetamine, for 60 min. These results showed that treatment with VA significantly ameliorated the methamphetamine-induced mitochondrial ROS formation, via its ROS scavenger activity.

### VA significantly inhibits mitochondrial GSH decline

**Fig. 4:**
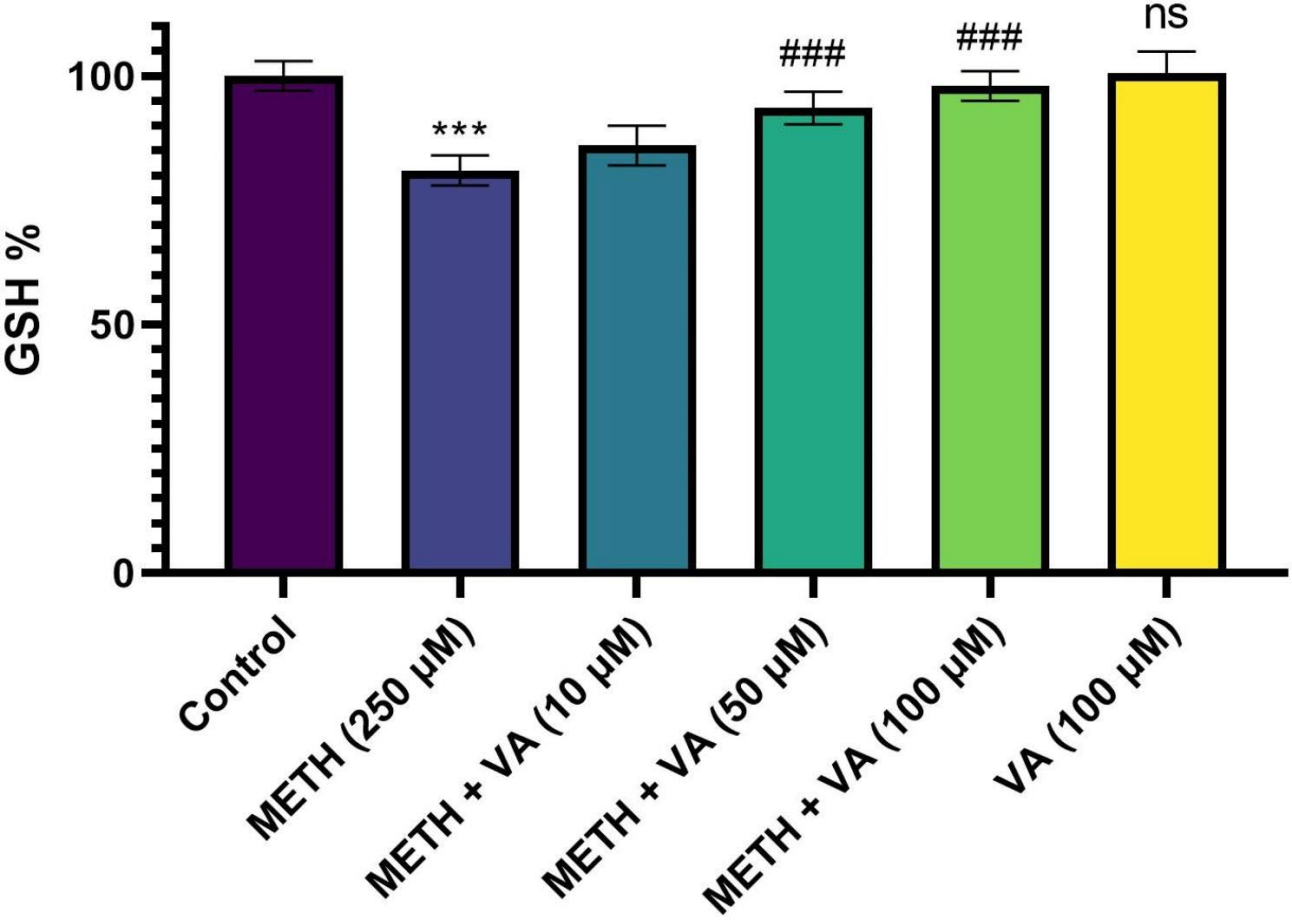
Protective effect of VA on GSH depletion caused by methamphetamine. Cardiac mitochondria were treated/cotreated with methamphetamine, methamphetamine plus various concentrations (10, 50 and 100 µM) of VA and VA (100 µM) alone for 60 min. The level of GSH was measured by the protocol of Ellman, as previously described in material and methods. Methamphetamine decreased GSH amounts in cardiac mitochondria. Treatment with various concentrations (50 and 100 µM) significantly inhibit the GSH depletion induced by methamphetamine. Data are presented as the mean ± SD. *** p < 0.001 versus untreated control; ### p < 0.001 versus methamphetamine group (one-way ANOVA followed by Tukey’s multiple comparison tests). GSH, glutathione; METH, methamphetamine; VA, Vanillic acid; SD standard deviation

Mitochondrial GSH as a key survival antioxidant in the health of mitochondria was assessed. As shown in Figs. 4 and 60 min incubation of cardiac mitochondria with methamphetamine resulted in a significant decline of the mitochondrial GSH level. Whereas, the decline of the level of mitochondrial GSH was significantly inhibited after incubation with VA (50 and 100 µM) and methamphetamine, for 60 min in cardiac mitochondria. These data confirm the important role of GSH in mitochondrial damage caused by methamphetamine and suggest that VA can inhibit methamphetamine-induced mitochondrial toxicity in cardiac mitochondria via its antioxidant potential. The mean absorbance of the non-exposed cardiac mitochondria was the reference value for calculating 100% mitochondrial GSH amounts.

### VA significantly decreases mitochondrial lipid peroxidation

**Fig. 5:**
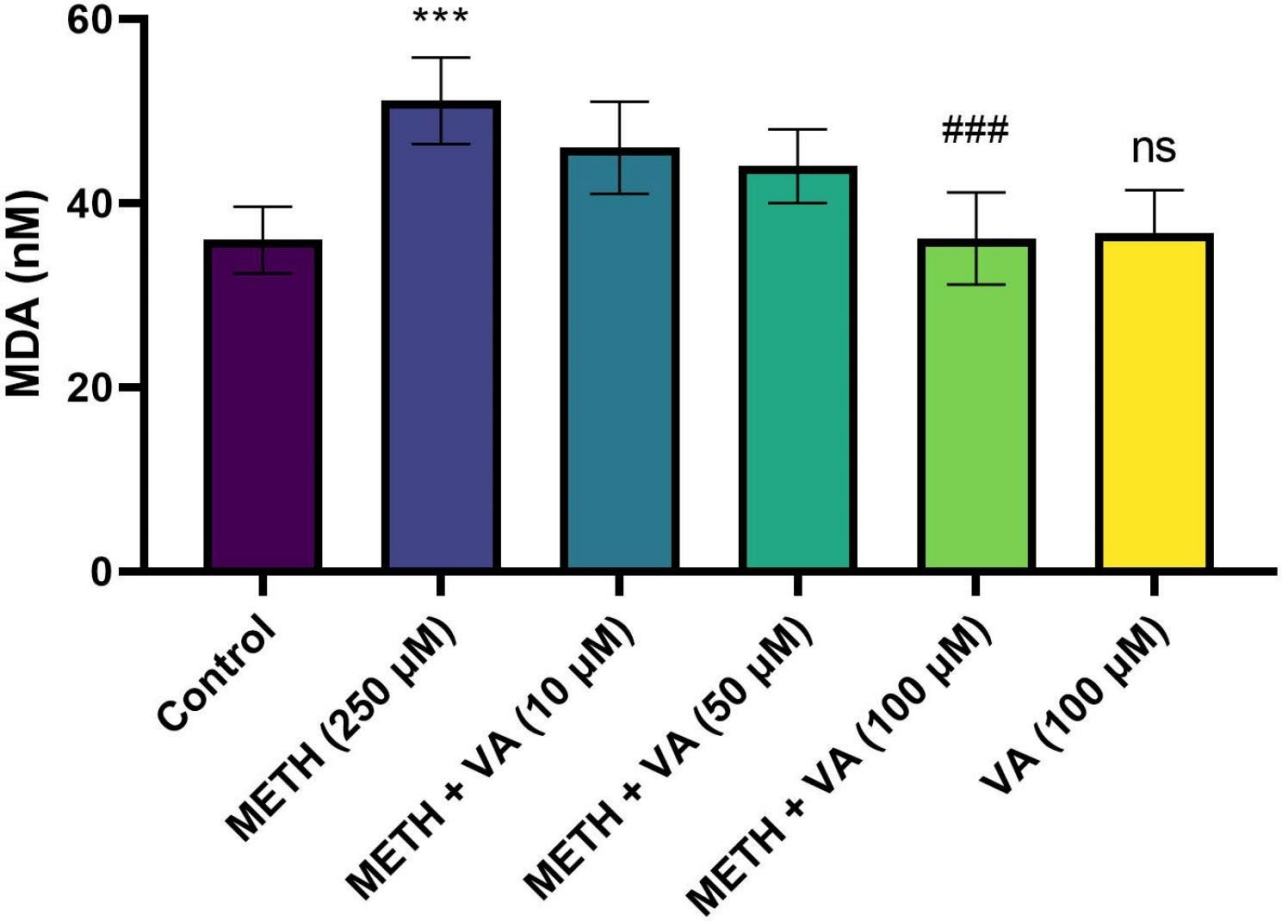
Inhibitory effect of VA on methamphetamine -induced mitochondrial lipid peroxidation. Cardiac mitochondria were treated/cotreated with methamphetamine, methamphetamine plus various concentrations (10, 50 and 100 µM) of VA and VA (100 µM) alone for 160 min. The level of MDA as indicator of mitochondrial lipid peroxidation was measured using TBARS test. Methamphetamine increased MAD contents in cardiac mitochondria. Treatment with VA (100 µM) significantly reduced methamphetamine-induced lipid peroxidation. Data are presented as the mean ± SD. *** p < 0.001 versus untreated control; ### p < 0.001 versus methamphetamine group (one-way ANOVA followed by Tukey’s multiple comparison tests). MDA, malondialdehyde; METH, methamphetamine; VA, Vanillic acid; SD standard deviation

MDA amounts as indicator of LPO were measured in cardiac mitochondria. In order to detect the effect of VA on the mitochondrial lipid peroxidation status in cardiac mitochondria, in presence of methamphetamine, MDA as indicator of lipid peroxidation was evaluated. As demonstrated in Fig. 5, after 60 min treatment of cardiac mitochondria with 250 µM methamphetamine, the level of MDA was significantly elevated to 51.12 ± 4.7 nM, compared to the untreated group 36.18 ± 3.5 nM. While, the level of MDA was significantly reduced after incubation with VA (100 µM) and methamphetamine, for 60 min in cardiac mitochondria. These data suggest that VA inhibits methamphetamine-induced mitochondrial toxicity in cardiac mitochondria through its antioxidant potential.

### VA significantly inhibits mitochondrial swelling

**Fig. 6:**
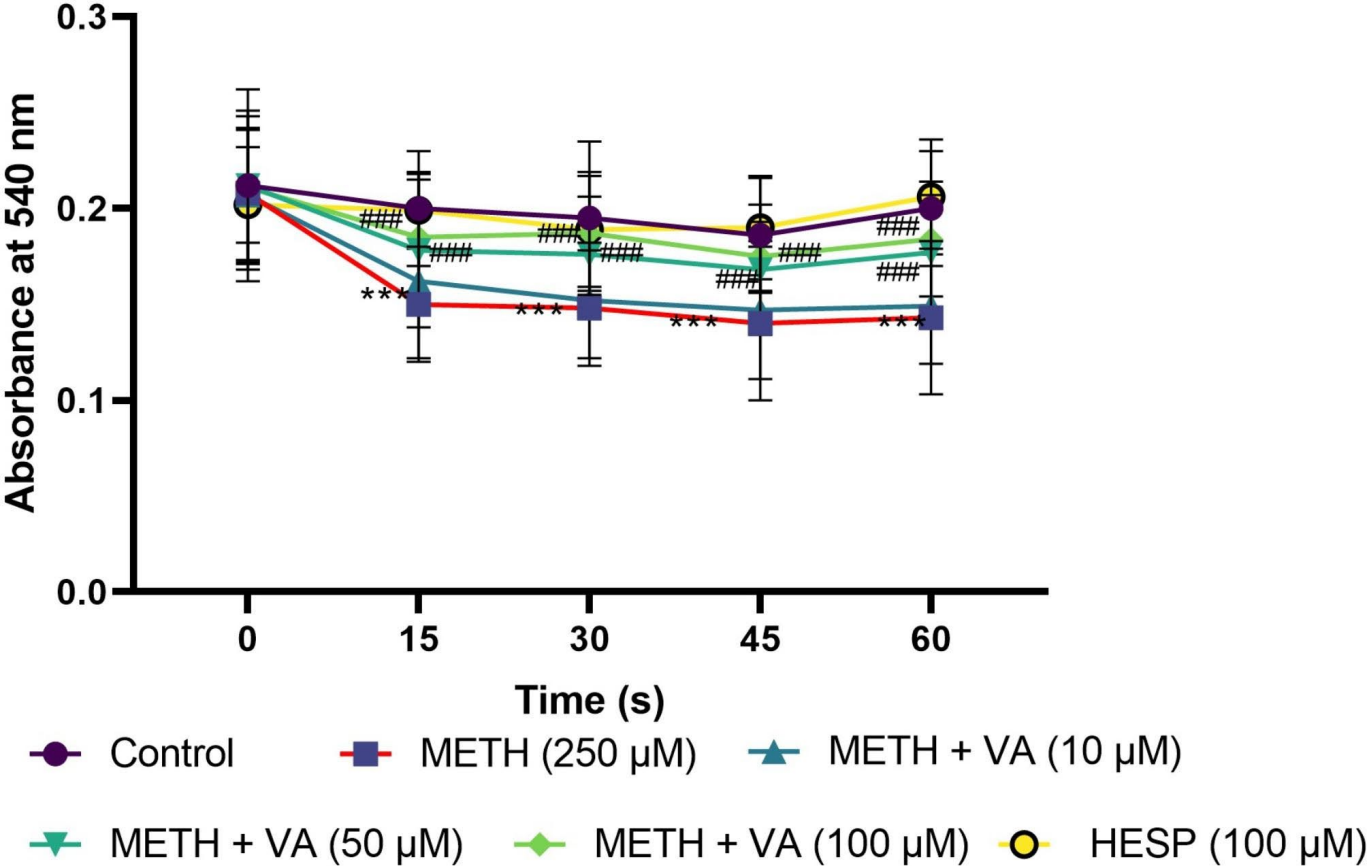
VA blocks methamphetamine-induced mitochondrial swelling. Cardiac mitochondria were treated/cotreated with methamphetamine, methamphetamine plus various concentrations (10, 50 and 100 µM) of VA and VA (100 µM) alone for 60 min. Mitochondrial swelling was measured by monitoring the absorbance at 540 nm by a microplate reader. Methamphetamine induced mitochondrial swelling in cardiac mitochondria. Treatment with various concentrations (50 and 100 µM) significantly block methamphetamine-induced mitochondrial swelling. Data are shown as the means ± SD of 5 experiments. *** p < 0.001 versus untreated control; ### p < 0.001 versus methamphetamine group (two-way ANOVA followed by Bonferroni’s test). METH, methamphetamine; VA, Vanillic acid; SD standard deviation

As a criterion of MMP collapse, mitochondrial swelling was measured by monitoring of the decreased absorbance at 540 nm. We measured the mitochondrial swelling as indicator of MPT pore (a mitochondrial mega channel) opening, which is closely associated with the subsequent release of mitochondrial lethal proteins and downstream activation of cell death events. As shown in Fig. 6, cardiac mitochondria began to swell quickly on exposure to methamphetamine and the absorbance decrease at 540 nm, as an indicator of mitochondrial swelling was observed. To further demonstrate that VA inhibit MPT pore opening, we used VA at different concentrations in the presence of methamphetamine. Intriguingly, VA (50 and 100 µM), when cotreated with methamphetamine alleviates the opening of MPT. These observations strongly indicate that VA inhibits MPT pore opening in mitochondria as ‘point of no return’ in mitochondrial toxicity.

## Discussion

Cardiac mitochondria are indispensable organelles in cardiomyocytes cardiac endothelial cells with various important roles in adenosine triphosphate (ATP) production, reduction/oxidation balance, regulation of intracellular homeostasis, proliferation, and cell death [27]. Any agent with toxic effects on cardiac mitochondrial function will result in alterations in ionic homeostasis, decreased production of energy, increased formation of harmful ROS cell injury and tissue death [27]. Today, cardiac mitochondria are a suitable tool to search the possible mitochondrial toxicity of drugs and chemicals [28]. Although the studies conducted on isolated mitochondria are not complete, but they can have several advantages compared to cell and animal studies. Some mitochondrial dysfunctions tests were not detected in animal and cellular investigations [29]. For example, most toxicity studies are done in adult young animals and ignored factors such as lack of adequate genetic diversity to induce idiosyncratic responses, absence of environmental factors, co-medication, insensitivity of histopathological experiments and for identifying mitochondrial dysfunction and having robust mitochondrial reserves in young tissues [29]. In addition, usually cell culture studies are not suitable to evaluate mitochondrial toxicity of various toxicant, due to high glucose in culture medium and shifting to glycolysis for energy production in this technique [29]. Therefore, the current study is devoted to investigating the direct toxicity of methamphetamine and the protective role of vanillic acid in cardiac mitochondria.

Due to a four-layer structure in the mitochondria (outer membrane, intermembrane space, inner membrane, and matrix), it is difficult for many molecules to enter these organelles. However, lipophilic and cationic molecules can enter to mitochondria by hydrophobic interactions and negative MMP [30]. The MMP is approximately − 180 mV and is 3- to 5-fold higher than the plasma membrane. Thus, lipophilic cationic molecules easily accumulate in the these organelles [31]. Methamphetamine as a cationic lipophilic molecule can diffuse to mitochondria and be retained by these organelles [32]. Accumulation of lipophilic cationic molecules in mitochondria eventually leads to destruction of the electrochemical gradient, increased production of ROS by complexes I and III, MMP collapse, mitochondrial GSH depletion, mitochondrial swelling, cytochrome c release, and a decrease in ATP generation [30]. Our result showed that methamphetamine exposure in cardiac mitochondria led to the MMP collapse. The MMP is the driving force of oxidative-phosphorylation and ATP generation. Alterations in MMP often reflect mitochondrial activity and commitment the mitochondria to membrane permeability changes, MMP collapse, ROS formation, oxidative stress, mitochondrial swelling and release of pro-apoptotic factors [33]. Our result confirmed that methamphetamine-induced MMP collapse in cardiac mitochondria is associated with SDH activity decline, ROS formation, GSH depletion, oxidative stress and mitochondria swelling. Consistent with our study, several mitochondrial, cellular and animal studies have demonstrated that mitochondrial dysfunction and induction of oxidative stress are the well- known mechanism for methamphetamine-induced cardiotoxicity [9–11, 34–39].

VA as a natural aromatic acid is used in the food industry as an additive agent. Due to phenolic nature of VA, it insulates the biological membrane and reduces lipid peroxidation in cells [40]. In the liver, VA can be produced by the oxidation of vanillin and crosses the blood-brain barrier [41]. Therefore, VA can enter cells and protect other biological membranes such as mitochondria, due to the structural role in the biological membrane and antioxidant potential. The current study is the first to provide evidence that VA directly reduced methamphetamine-induced mitochondrial toxicity in cardiac mitochondria. We found that VA as well-known antioxidant with cardioprotective properties is able to reduce mitochondrial toxicity induced by methamphetamine, which is associated with the increase of SDH activity, inhibition of MMP collapse and the decrease of ROS formation, oxidative stress and mitochondria swelling. Consistent with our study, several studies have demonstrated that VA can reduce mitochondrial dysfunction, oxidative stress and cardiotoxicity. These studies have shown that VA ameliorates cardiotoxicity with improvement mitochondrial function, reduction in ROS production, aggravation of antioxidative status, scavenging free radicals and inhibition of lipid peroxidation [21, 42–47]. In addition to the beneficial effects of VA on cardiac mitochondria, this phenolic compound has promising effects on other mitochondria in different cells and tissues. Ay has reported that VA can serve as a potential neuroprotective agent via expression of genes related to mitochondrial biogenesis in neuronal cells [48]. It has reported that VA can bypass mutations of *COQ6*, as gene involving in synthesis Coenzyme Q (CoQ) [41]. CoQ as a redox-active lipid and acts as an electron carrier in the mitochondrial respiratory chain, an essential antioxidant and a cofactor of other mitochondrial dehydrogenases [41]. Therefore, VA is promising compound for inhibition of mitochondrial dysfunction due to CoQ deficiency related to COQ6 mutations [41]. Our study, consistent with previous studies demonstrate that VA could potentially serve as an accessible, promising and mitochondrial protective agent against methamphetamine-induced cardiotoxicity.

Our results in the current study have proven that VA protects cardiac mitochondria against methamphetamine-induced mitochondrial toxicity, via stabilizing mitochondrial membrane potential, reducing the formation of ROS, limiting the opening of MPT pore, decreasing oxidative, and inhibiting GSH depletion. This study suggested that VA has the potential protective against methamphetamine-induced mitochondrial toxicity. It seems that this natural compound found in food be benefit for medical uses such as mitochondria-related cardiovascular dysfunctions. As limitation of the current work, the beneficial effects of VA on methamphetamine-induced cardiotoxicity should be further searched by using cellular, animal and clinical trial studies.

## Data Availability

The data and materials are available from the corresponding author upon reasonable request.
